# Prediction of the Biogenic Amines Index of Poultry Meat Using an Electronic Nose

**DOI:** 10.3390/s19071580

**Published:** 2019-04-01

**Authors:** Wojciech Wojnowski, Kaja Kalinowska, Tomasz Majchrzak, Justyna Płotka-Wasylka, Jacek Namieśnik

**Affiliations:** 1 Department of Analytical Chemistry, Faculty of Chemistry, Gdańsk University of Technology, 80-233 Gdańsk, Poland; tomasz.majchrzak@pg.edu.pl (T.M.); juswasyl@pg.edu.pl (J.P.-W.); chemanal@pg.gda.pl (J.N.); 2 Singapore-MIT Alliance for Research and Technology, Center for Environmental Sensing and Modeling, Singapore 138602, Singapore

**Keywords:** electronic nose, meat, neural networks, DLLME-GC-MS, headspace analysis, electronic olfaction, biogenic amines

## Abstract

The biogenic amines index of fresh chicken meat samples during refrigerated storage was predicted based on the headspace analysis using an electronic nose equipped with an array of electrochemical sensors. The reference biogenic amines index values were obtained using dispersive liquid–liquid microextraction–gas chromatography–mass spectrometry. A prototype electronic nose with modular construction and a dedicated sample chamber was used to rapidly analyze the volatile fraction of chicken meat samples, with a single measurement time of five minutes. Back-propagation artificial neural network was used to estimate the biogenic amines index of the samples with a determination coefficient of 0.954 based on ten-fold stratified cross-validation. The results indicate that the determination of the biogenic amines index is a good reference method for studies in which the freshness of meat products is assessed based on headspace analysis and fingerprinting, and that the described electronic device can be used to assess poultry meat freshness based on this value with high accuracy.

## 1. Introduction

The use of the electronic nose technique has several advantages in regard to the assessment of meat products’ freshness. It is non-destructive, allows for rapid analysis (several minutes), and the cost of the instrument is relatively low, especially when compared to chromatographic techniques and mass spectrometry [1,2]. Furthermore, since headspace analysis of meat samples using electronic olfaction is based on a holistic ‘fingerprinting’ approach, the results are more closely related to the way humans perceive spoilage through sensory analysis. For this reason, a number of studies have focused on the use of these devices for the assessment of shelf life and freshness of meat stored under refrigeration [3,4,5,6].

However, the sensory quality of meat ‘freshness’ is rather subjective, and as such leaves much to be desired in the context of the evaluation of meat spoilage based on the analysis of the sample’s volatile fraction. One obvious threshold would be the shelf-life provided by the distributor, with classification simply based on the duration of storage. In such an approach it is difficult to account for independent variables such as storage conditions, meat origin or even the composition of feed given to the animals [7]. The determination of the total viable bacteria is a common reference technique [8], despite it being relatively labor-intensive and time-consuming (up to 72 h). It is also less applicable to the evaluation of frozen or refrigerated products, as it favors the proliferation of mesophilic bacteria, as opposed to psychrotrophic organisms which are better adapted to lower temperatures [9]. Moreover, the presence of bacteria alone is not necessarily a clear indication of spoilage, and bacteriological analysis cannot be used to assess the effects of chemical spoilage, which is an important factor in the overall deterioration of meat products [10]. As an alternative, several meat freshness indices based on the concentration of biogenic amines have been proposed [11,12]. In particular, the biogenic amines index (BAI) [13] is the total concentration of tyramine, putrescine, cadaverine, and histamine in a sample, and the concentration of these amines in the sample has been shown to be a reliable indicator of poultry meat freshness [14]. Biogenic amines (BAs) can be determined in meat samples using several instrumental analytical techniques, with high-performance liquid chromatography (HPLC) and gas chromatography (GC) being among the most popular methods [15]. The latter in particular, when coupled with adequate sample preparation and derivatization method such as dispersive liquid–liquid microextraction–gas chromatography–mass spectrometry (DLLME-GC-MS), has the potential for application in routine tests due to the relatively short time of a single analysis and high sensitivity [16].

The use of the BAI value as a reference in a holistic headspace analysis of chicken meat has several advantages. Firstly, the determination of BAs using instrumental methods is significantly faster than the bacteriological analysis. Moreover, the BAI score is an objective, absolute value that can be easily referenced, and is a continuous variable which enables the use of regression machine learning models. Artificial neural networks (ANN) in particular seem to be applicable in such a scenario due to their flexibility and potential for gradual re-training with the increasing data sets, which is to be expected in real-world deployments.

The most straightforward approach to chicken meat freshness assessment using electronic olfaction is to predict discretized features such as duration of storage [17,18,19] or threshold values (e.g., 6.0 log_10_ CFU/g (CFU—colony forming units)) [20]. Electronic noses have also been previously used to predict non-discretized values of the bacterial population or total volatile basic nitrogen in chicken samples based on supervised machine learning algorithms [21,22]. As an alternative approach, in this study we propose to relate the electronic nose measurements to the concentration of spoilage indicators in the samples, as determined using a relatively rapid instrumental analytical method. Furthermore, most other studies on the use of electronic olfaction for poultry freshness evaluation have involved metal oxide semiconductor (MOS) sensors. These have several advantages such as robustness, low cost, and sensitivity to a wide range of chemicals; however, due to high power consumption and susceptibility to humidity, they are not ideal for the use in portable and hand-held devices. Instead, we propose the use of modern electrochemical sensors with low energy consumption and resilience towards humidity changes [23], which might facilitate the development of compact, battery-powered units for field use. The aim of this study was to assess the possibility to predict the value of the biogenic amines index of fresh chicken breast muscle samples stored under refrigeration using an electronic nose device. The electronic nose was equipped with an array of electrochemical gas sensors and was purposefully configured with a headspace analysis of meat samples in mind. The BAI values were determined using a validated DLLME-GC-MS method for quantitative analysis of biogenic amines in animal muscle samples. The predictions were made using a model based on artificial neural networks.

## 2. Materials and Methods

### 2.1. Samples

Samples of fresh chicken breast muscle (*pectoralis major*) were obtained from various local distribution centers in Gdańsk, Poland in order to test the robustness of the analysis with respect to samples sourced from different outlets. The birds were slaughtered and dismembered one day prior to the first day of the analysis of each batch, and the meat was stored at 2.4 °C. On the first day of analysis, 0.5 kg of breast muscle meat was ground and transported under refrigeration to the laboratory, where samples of 4 g each, intended for e-nose and DLLME-GC-MS analysis, were placed in 20 cm^3^ glass headspace vials and covered with food cling film made of polyvinylidene chloride to emulate real storage conditions. Samples were then stored at 4 °C. On each day of the experiment, 9 samples from each batch were analyzed using the electronic nose, and 3 samples using GC-MS. Prior to the electronic nose analysis, the headspace vials were sealed with a metal cap lined with a silicon-PTFE membrane. Samples from each batch were analyzed for five consecutive days since it had previously been established that beyond that the consumers deem poultry meat as unacceptable based on sensory analysis alone [24]. From each batch 12 samples were analyzed every day, with a total of 60 samples per batch and 180 samples throughout the experiment.

### 2.2. Electronic Nose

**Figure 1:**
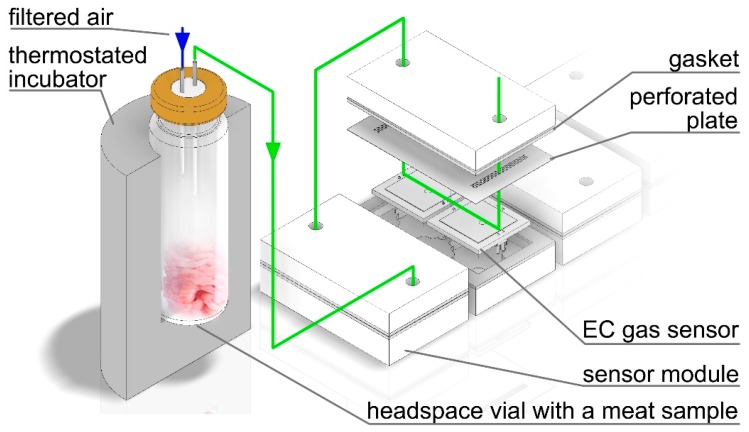
A schematic representation of the sampling with an exploded view of a single sensor module containing two electrochemical gas sensors and a perforated plate to stabilize the gas flow.

The prototype electronic nose used in this study had been developed in order to analyze the volatile fraction of food products and, in particular, meat. It is equipped with an array of eight electrochemical gas sensors, each partially selective to a different group of chemical compounds. The sensors were chosen based on their longevity (up to 10 years lifespan) and resilience to changes in relative humidity [25]. The DGS-CO 968-034, DGS-EtOH 968-035, DGS-H_2_S 968-036, DGS-NO_2_ 968-037, DGS-SO_2_ 968-038, and DGS-RESPIRR 968-041 volatile organic compounds (VOC) sensors were manufactured by SPEC Sensors, Newark, CA, USA, while the 2E 50 tert-butyl mercaptan (TBM) and 3E 100 SE(NH_3_) sensors were manufactured by City Technology, Portsmouth, UK. They have been factory calibrated to the designated chemical compounds [26,27]; however, the complex nature of the headspace of meat samples precludes their use for qualitative or quantitative determination. Instead, they were used for holistic fingerprinting. The device is also equipped with an additional humidity sensor to monitor the changes of relative humidity, and a pressure sensor to signal blockages in the pneumatic assembly. The sensors are paired in four interchangeable blocks connected in-line. The samples are incubated in a thermostated block which can accommodate the 20 cm^3^ headspace vials. Ambient air passed through a VOC filter is used both as a carrier gas when directed through the sample’s headspace as shown in Figure 1, and for purging the sensors and pneumatic setup after each subsequent analysis. The flow rate during both purging and sampling was fixed at 100 cm^3^min^−1^. The raw signals from the EC sensors are processed by an integrated microprocessor and the device’s operation is controlled via a PC-class computer with dedicated software and automatically compensated for temperature. During each analysis, the baseline for the carrier gas was registered and automatically subtracted from the sensors’ responses in the sampling mode. After prolonged periods of disuse, the sensors require a zero calibration and stabilization upon powering the device. This is ensured by sampling clean air for at least an hour in continuous mode. For a more detailed description of the device’s construction and operation, the reader is directed to a previous article [28].

The incubation temperature was set to 37 °C in order to facilitate the transfer of volatile chemical compounds into the sample’s headspace whilst avoiding the denaturation of protein. A single analysis consisted of three stages: 100 s of incubation, during which the sensors were purged with ambient air, followed with 100 s of sampling with the carrier gas directed through the sample’s headspace (Figure 1) and 100 s of subsequent purging, with a total of 300 s per sample.

### 2.3. Dispersive Liquid–Liquid Microextraction Combined with Gas Chromatography–Mass Spectrometry

The development and validation of the DLLME-GC-MS analytical method for the in situ determination of biogenic amines in fresh animal muscle samples has been described in detail elsewhere [29]. The use of this method enables rapid derivatization and extraction of the BAs. It is characterized by good recoveries, intra- and inter-day repeatability, and linearity. Its limits of quantification range from 0.009 μg·g^−1^ to 0.029 μg·g^−1^ for particular BAs.

In brief, an appropriate volume of sodium hydroxide solution was added to each sample, and they were homogenized, centrifuged, and sonicated. This was followed by another centrifugation, and the supernatant was made up to 50 cm^3^, with 5 cm^3^ of the supernatant subjected to derivatization and extraction procedure. The 4 g samples of ground chicken breast muscle were spiked with internal standard (50 mm^3^ aquatic solution at 100 mgdm^−3^) and a mixture of pyridine: HCl (1:1, v/v), methanol (210 mm^3^), chloroform (300 mm^3^), and isobutyl chloroformate (100 mm^3^) was rapidly injected into the sample vial and kept for 10 min. Afterwards, 1 cm^3^ of chloroform was added and the sample was hand-shaken for 5 min. The bottom layer of the separated solution was sampled (200 mm^3^) for subsequent GC-MS analysis.

All the reagents and standards were obtained from Sigma Aldrich (Steinheim, Germany) and were of analytical grade. The BAs (cadaverine, putrescine, histamine, and tyramine) were in the form of hydrochloride salts, and the standard solutions (1.0 mg cm^3^) were prepared by dissolution in deionized water and stored in silanized vials sealed with PTFE-lined caps. The ultrapure water was obtained using a Milli-Q water purification system (Millipore, Bedford, MA, USA).

The analysis was performed using the Agilent 7890A gas chromatograph (Agilent Technologies, Santa Clara, CA, USA) with the Zebron ZB-5MS capillary column (30 m × 0.25 mm I.D., 0.25 μm film thickness) (Phenomenex, Torrance, CA, USA), and coupled with the Agilent 5975C mass-selective detector with an electron ionization chamber (Agilent Technologies, Santa Clara, CA, USA). The column is well-suited for high-sensitivity GC-MS analyses and is recommended for use with amines. The injection was performed in splitless mode at 32 psi and 240 °C. Helium at 30 psi was used as carrier gas. The data was collected using Agilent ChemStation software.

### 2.4. Statistical Analysis and Machine Learning

The output of the e-nose device in the form of comma-separated values was processed using the Orange Python package. The sensor response values were normalized (centered by mean and, since they do not follow the normal distribution, scaled by span). Instead of assigning an average BAI value to batches of 9 samples analyzed using the electronic nose, the three BAI values obtained for each batch during the DLLME-GC-MS analysis were instead assigned to sub-groups of three e-nose measurements. This was not done due to an affinity between these particular samples since they all came from the same batch, but to prevent overfitting during the subsequent machine learning stage. The missing data (concentrations of particular BAs below the limit of quantitation (LOQ)) were substituted with the limit of detection (LOD)/3 values. Four sensors, the responses of which had the greatest impact on the estimation of the BAI, were then selected based on the analysis of variance using the RReliefF algorithm [30]. Since the number of variables was relatively low compared to the number of instances, there was no need to further reduce the dimensionality of the data set using, for example, the principal component analysis. The machine learning regression model was based on a back-propagation artificial neural network and was performed using the SciKit Learn v.0.20.2 Python package. The network was comprised of four nodes in the input layer, two hidden layers each with four nodes, and a single node in the output layer, with a stochastic gradient-based optimizer (Adam). Rectified linear unit function (ReLu) was used for activation, and the regularization strength (learning rate) was set to 0.01. The training was iterated until convergence was reached. The model was validated using a 10-fold stratified cross-validation. Additionally, 75% of the data set, randomly selected, was used for training, and the remaining 25% of the dataset was used for testing, with the random sampling/training/testing repeated 10 times, and the scores averaged.

## 3. Results and Discussion

### 3.1. Determination of the Biogenic Amines Index

Based on the DLLME-GC-MS analysis it can be observed that the marked increase of the BAI value after the second day of refrigerated storage is mostly due to the increased concentration of cadaverine. The appearance of poultry meat spoilage indicators after the second day of refrigerated storage is supported by both sensory analysis [24] and bacteriological analysis [31], and also corroborates with the results obtained earlier using the same method [29]. Although in the referenced study the samples were stored under slightly different conditions (different containers). The rapid progression of spoilage is partly the result of the bacteria entering the logarithmic growth phase after the initial lag phase, and in the case of BAs in poultry meat samples occurs earlier than in other types of meat due to the presence of shorter protein chains, which leads the generation of amino acid precursors of amines [32]. An example of the results of the determination of the four BAs in the poultry meat samples is shown in Table 1, while the averaged results for all three batches are shown in Table 2. Since the BAs were determined in the sample, while the e-nose measurements are limited to the sample’s headspace in which these amines are likely present at concentration levels below the sensors’ LOD due to their limited volatility, the post-hoc analysis of the correlation between the results of both measurements was not undertaken.

### 3.2. Electronic Nose Measurements

**Figure 2:**
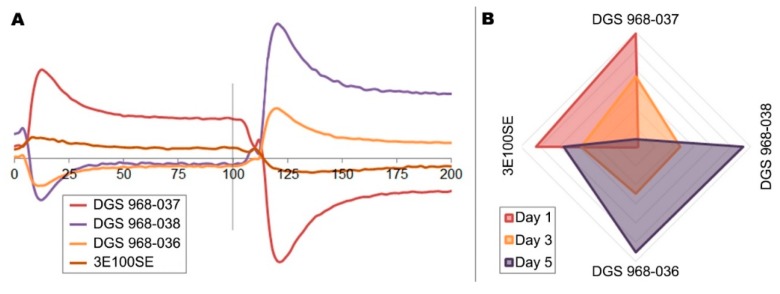
(**A**) Response of the four electrochemicalsensors to the change from purging to sampling mode when analyzing a sample of poultry meat after 5 days of refrigerated storage; the change of sampling modes occurs after 100 s, and the axis denotes time expressed in seconds. The spikes are the result of temporary pressure changes caused by valve operation; (**B**) radar plot of the normalized measurements of the four EC sensors when analyzing the headspace of poultry meat samples on the first, third, and fifth day of the experiment; days 2 and 4 were omitted for clarity.

The main advantage of the e-nose technique compared to other instrumental methods of food freshness assessment, apart from its relatively low cost, is the short time of a single analysis. In order to capitalize on this advantage, the entire measurement cycle for a single sample, including incubation, was constrained to five minutes. Based on the analysis of variance it was determined, that the response signals of the DGS 968-037, DGS 968-038, DGS 968-036, and 3E100SE sensors had the greatest impact on the regression with respect to BAI. This confirms previous findings [28] and is also to be expected since the increase of the concentration of nitric and sulfuric compounds is generally associated with meat spoilage. The averaged sensor response values after subtracting the background signal are listed in Table 2. It is important to note that the particular sensors are only partially selective to the indicated chemical compounds, and so their response value is not directly correlated with the concentration of these particular volatiles in the samples headspace. The response signals of the four EC gas sensors during sampling and purging, converted by the integrated analog to digital converter microprocessor, are shown in Figure 2. The initial spikes of the signal in each sampling mode are the result of pressure change caused by the turning of the ball valve which switches between the purging and sampling pneumatic circuits, and by the passing reaction to the humidity change due to exposure to the sample’s headspace. For the actual readout, the sensor responses during the final 10 s of each mode were averaged after the steady state of the signal was reached. The humidity in the sampling mode increased uniformly to approximately 81% and did not affect the sensors’ response at the data registration stage.

### 3.3. Multivariate Statistical Analysis and ANN Regression

**Figure 3:**
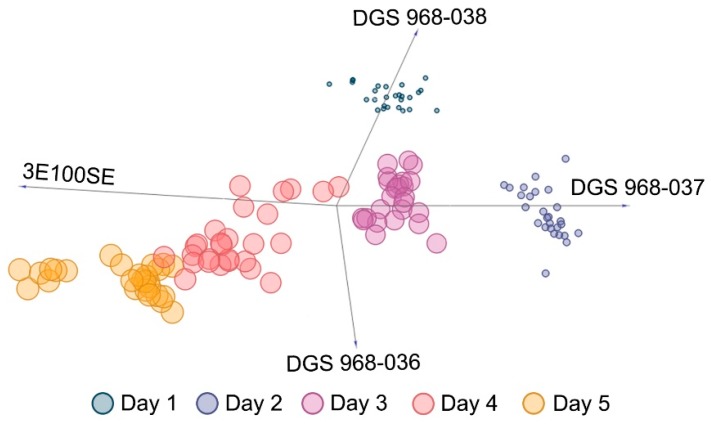
A projection of the four input variables for the training of the regression model; the size of the data points is directly proportional to the corresponding BAI value.

**Figure 4:**
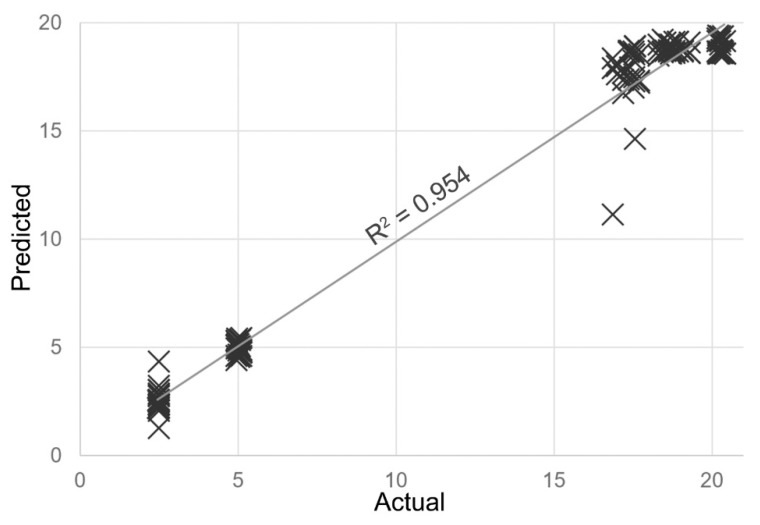
A plot of the BAI values determined in poultry meat samples stored over a period of 5 days using DLLME-GC-MS and the corresponding BAI values estimated with a trained artificial neural networks (ANN) model based on the results of headspace analysis using an electronic nose device.

As mentioned in the previous sub-section, based on the analysis of variance using the RReliefF algorithm it was determined that the response signals of the DGS 968-037, DGS 968-038, DGS 968-036, and 3E100SE sensors contributed the most to the prediction of BAI values based on the results of e-nose analysis. The RReliefF scores for particular sensors were as follows: 3E 100 SE (0.287); DGS 968-036 (0.147); DGS 968-037 (0.145), DGS 968-038 (0.139); 2E 50 (0.132); DGS 968-034 (0.131); DGS 968-041 (0.120); DGS 968-035 (0.091). A FreeViz linear projection of the four variables, which shows their contribution to the separation of data instances into different classes [33], is shown in Figure 3. A back-propagation artificial neural network was then trained to estimate the BAI values based on the output signals of these four electrochemical sensors registered using an electronic nose. Based on the validation results, the coefficient of determination was 0.954 (*p* < 0.01), as shown in Figure 4, and the root mean square error (RMSE which might be interpreted as the standard deviation of the unexplained variance) was 1.65. These are satisfactory results, and only a single instance of gross underestimation was obtained during the cross-validation. The average classification accuracy when testing on the separate test set was 0.947 (*p* < 0.01), with a RMSE of 1.66. Due to the rapid increase of the BAI value after the second day of refrigerated storage of fresh poultry meat samples, no intermediate BAI values were determined. This is due to the nature of the chemical and biological spoilage of chicken meat; however, it did not adversely affect the performance of the model. The reason for choosing the ANN model and not one of the other regression machine learning models, such as support vector machines (SVM) or random forest (RF), is that in the future real application of it will scale well with incremental re-training as additional data points are added during routine operation. With just four nodes in the input layer, the proposed architecture of the network (two hidden layers each with four nodes) will likely be able to perform well even with a significant increase of the size of the dataset without drastically increasing the computational power demands.

## 4. Conclusions

The determination of the biogenic amines index is a good reference method for studies in which the freshness of meat products is assessed based on headspace analysis and fingerprinting. Using the results of DLLMA-GC-MS analysis it is possible to validate the results of electronic nose measurements and provide a basis for a regression machine learning model with higher real-world application potential than discrete classification approaches. The prototype modular electronic nose equipped with electrochemical sensors, coupled with artificial neural networks-based machine learning algorithm, was capable of producing good classification accuracy. Despite the fact that the samples were sourced from different distributors the results were reproducible, demonstrating that the proposed approach is to an extent resilient to unaccounted-for independent variables. Based on the results it can be also concluded that electronic noses with sensor arrays and construction tailored to the specific application of meat freshness evaluation can be used to obtain reliable results rapidly and at a relatively low cost, and that the potential commercial use of this type of device is not a far-fetched idea.

## Figures and Tables

**Table 1:** Concentration (mg/kg, average ± mean square error (MSE)) of cadaverine (CAD), histamine (HIST), putrescine (PUT), and tyramine (TYR) in three samples from the first batch of chicken breast muscle, analyzed daily, and the corresponding value of the biogenic amines index (BAI).

Sample	Day	CAD	HIST	PUT	TYR	BAI
1	1	<LOQ ^1^	1.484 ± 0.045	0.991 ± 0.022	<LOQ	2.475
	2	1.921 ± 0.037	1.434±0.037	1.011±0.045	0.567±0.021	4.933
	3	8.11 ± 0.52	4.29 ± 0.15	1.134 ± 0.023	3.32 ± 0.16	16.85
	4	9.01 ± 0.61	4.09 ± 0.18	1.354 ± 0.030	3.83 ± 0.19	18.28
	5	10.45 ± 0.28	3.84 ± 0.14	1.799 ± 0.031	4.16 ± 0.20	20.25
2	1	<LOQ	1.472 ± 0.040	0.979 ± 0.018	<LOQ	2.451
	2	1.924 ± 0.035	1.429 ± 0.032	1.011 ± 0.044	0.577 ± 0.009	4.941
	3	8.58 ± 0.23	4.27 ± 0.15	1.128 ± 0.027	3.19 ± 0.18	17.17
	4	9.11 ± 0.63	4.11 ± 0.19	1.404 ± 0.034	3.79 ± 0.20	18.41
	5	10.51 ± 0.31	3.76 ± 0.10	1.812 ± 0.031	4.13 ± 0.19	20.21
3	1	<LOQ	1.481 ± 0.047	0.987 ± 0.021	<LOQ	2.468
	2	1.927 ± 0.030	1.424 ± 0.034	1.015 ± 0.047	0.570 ± 0.011	4.936
	3	8.31 ± 0.51	4.21 ± 0.16	1.129 ± 0.022	3.32 ± 0.18	16.97
	4	9.20 ± 0.61	4.16 ± 0.16	1.414 ± 0.030	3.76 ± 0.22	18.53
	5	10.47 ± 0.33	3.90 ± 0.17	1.832 ± 0.030	4.19 ± 0.19	20.39

^1^ LOQ—limit of quantitation.

**Table 2:** Averaged EC sensor response values ± standard deviationand corresponding averaged biogenic amines index (BAI) values obtained usingdispersive liquid–liquid microextraction–gas chromatography–mass spectrometry (DLLME-GC-MS) for three batches of chicken breast muscle refrigerated over a period of five days.

Batch	Day	DGS 968-037	DGS 968-038	DGS 968-036	3E100SE	BAI
1	1	−415.0 ± 11.2	391.4 ± 12.8	126.8 ± 4.8	−143.0 ± 8.1	2.465
	2	−554.8 ± 10.9	527.2 ± 13.3	169.0 ± 4.0	−280.2 ± 7.1	4.937
	3	−511.6 ± 19.9	483.0 ± 16.6	160.0 ± 4.2	−203.2 ± 9.4	17.00
	4	−565.6 ± 15.7	540.2 ± 12.6	178.0 ± 5.9	−189.0 ± 5.9	18.41
	5	−652.4 ± 6.9	621.6 ± 3.3	202.2 ± 2.6	−179.8 ± 5.8	20.28
2	1	−458.4 ± 4.6	435.2 ± 6.4	139.2 ± 1.4	−175.2 ± 5.1	2.472
	2	−589.8 ± 4.7	556.0 ± 4.1	179.8 ± 2.6	−306.6 ± 5.3	5.070
	3	−561.4 ± 6.9	542.4 ± 6.7	178.0 ± 3.5	−234.2 ± 4.3	17.58
	4	−634.4 ± 6.4	603.0 ± 6.7	195.2 ± 2.9	−205.0 ± 3.1	18.91
	5	−662.0 ± 5.2	640.4 ± 7.2	210.4 ± 2.0	−189.6 ± 2.8	20.31
3	1	−453.0 ± 10.7	426.0 ± 11.0	136.4 ± 2.5	−182.6 ± 6.1	2.471
	2	−610.4 ± 4.5	571.2 ± 8.2	187.4 ± 4.2	−323.2 ± 2.3	5.003
	3	−595.2 ± 9.5	570.8 ± 6.8	190.4 ± 4.0	−255.8 ± 9.7	17.43
	4	−648.4 ± 7.3	618.8 ± 5.1	204.0 ± 2.8	−201.4 ± 6.3	18.85
	5	−665.4 ± 1.6	641.4 ± 4.9	213.2 ± 1.3	−159.4 ± 4.7	20.20
